# Multiscale Mechanisms Underlying the Invasion Success of *Pomacea canaliculata*: A Review

**DOI:** 10.3390/biology15100747

**Published:** 2026-05-08

**Authors:** Xiaoyang Bi, Yaxin Ren, Xu Kuang, Mengping Zhang, Zheng Zhao, Tao Zhu, Guikui Chen

**Affiliations:** 1 School of Life Sciences and Engineering, Henan University of Urban Construction, Pingdingshan 467036, China; 2 College of Natural Resources and Environment, South China Agricultural University, Guangzhou 510642, China

**Keywords:** apple snail, biological invasion, environmental tolerance, climate warming, rapid evolution, niche adaptation

## Abstract

*Pomacea canaliculata*, listed among the world’s 100 worst invasive alien species, poses serious threats to rice production and freshwater ecosystems. This review synthesizes current research in physiological ecology, molecular genetics, and invasion ecology to examine its invasion success from a multiscale mechanistic perspective. *P. canaliculata* exhibits broad environmental adaptability at physiological, molecular, and behavioral levels. These adaptations include seasonal cold tolerance, drought-induced dormancy and post-dormancy recovery, acclimation to both freshwater and brackish environments, and tolerance to a range of pollutants and pesticides, including evidence of toxicant-induced hormesis. The species also shows pronounced phenotypic plasticity in growth, reproduction, and resource utilization. Genomic plasticity, multiple introduction events, and introgressive hybridization with closely related species further enhance its evolutionary potential and dispersal capacity. In addition, *P. canaliculata* displays behavioral adaptations such as learning and alarm responses. The synergistic interaction of these multilayered adaptive mechanisms underpins the global invasion success of this species. This review also identifies key uncertainties in current research and emphasizes the need for greater integration of multi-omics approaches, long-term monitoring of population dynamics in hybrid zones, and experimental studies addressing the interactive effects of multiple stressors, with the ultimate aim of improving invasion risk prediction and management.

## Simple Summary

The apple snail (*Pomacea canaliculata*) is among the world’s most destructive invasive animals, causing severe damage to rice production and freshwater ecosystems. This review examines the mechanisms underlying its invasion success and the challenges associated with its control. By integrating evidence from physiology, genetics, and behavior, we show that the snail can withstand a wide range of environmental stresses. It tolerates seasonal cold, enters dormancy during drought and resumes activity upon rehydration, survives in both freshwater and mildly brackish habitats, and exhibits tolerance to certain pollutants and control agents. The species also displays substantial flexibility in growth, reproduction, and resource use under changing environmental conditions. In addition, genomic variation, repeated introductions, and introgressive hybridization with closely related species may further enhance its adaptive capacity and invasion potential. Behavioral traits, including learning ability and responses to danger signals, also contribute to its ecological success. This review also identifies important gaps in current knowledge and calls for more long-term and combined studies. A deeper understanding of these adaptive mechanisms will support improved early warning, management, and protection of agricultural production and freshwater ecosystems.

## 1. Introduction

In the context of global environmental change, biological invasions have become one of the most pressing ecological challenges of the modern era, profoundly altering ecosystem structure and function [1]. Biological invasion refers to the process by which organisms expand beyond their native range, establish self-sustaining populations, and spread in novel environments through natural dispersal or human-mediated introduction [2]. By overcoming geographic and reproductive barriers, invasive species can severely threaten native biodiversity, disrupt ecosystem functioning, and cause substantial ecological and economic damage [3]. In particular, the introduction of nonnative species can impair ecosystem services, reduce agricultural productivity, and even compromise land abandonment as a conservation strategy [4]. Over recent decades, biological invasions have been estimated to cause at least USD 1.288 trillion in global economic losses, with an average annual cost of approximately USD 26.8 billion. By 2017, the annual cost had risen to an estimated USD 162.7 billion, although these figures are widely considered substantial underestimates. Notably, invasion-related costs have increased at an approximate rate of threefold per decade [5]. For apple snails alone, agricultural losses in Southeast Asia are especially severe: annual rice production losses in the Philippines, Thailand, Indonesia, and Viet Nam are estimated at USD 2.138 billion [6].

As a widely traded and globally popular ornamental aquatic species, the apple snail, native to South America, has been extensively introduced beyond its native range [6]. Through human-mediated transport and intentional or accidental release, it has dispersed across Asia, Africa, Europe, Oceania, and the Americas [6,7]. As a highly adaptable, semi-sessile amphibious gastropod, it can cause crop yield losses of up to 50% [7]. The family Ampullariidae is naturally distributed throughout tropical and subtropical humid regions worldwide and comprises approximately 10 genera and 120 species. Among them, species of the genus *Pomacea* are the most diverse and intensively studied [8]. Within this genus, *P. canaliculata* and *P. maculata* exhibit the greatest invasive potential and inflict the most serious damage [9]. Owing to its substantial environmental impact, *P. canaliculata* has been included by the International Union for Conservation of Nature (IUCN) Invasive Species Specialist Group among the “100 Worst Invasive Alien Species”. Although the majority of available studies focus on *P. canaliculata*, some also involve *P. maculata*; therefore, many of the conclusions drawn in this review may be broadly applicable to both species.

Biological invasion is generally regarded as a dynamic, multistage process comprising introduction, transport, release, colonization, establishment, and spread, with success or failure possible at each stage [10,11]. Phenotypic traits are widely recognized as key determinants of invasion success throughout this process [3]. Considerable research has investigated the traits that contribute to the invasion success of *P. canaliculata* at different stages [9]. During introduction and transport, attention has focused on its tolerance to poor water quality, hypoxia, and physical disturbance [12,13]. During colonization and establishment, studies have emphasized its high reproductive output, broad diet, wide ecological niche, and ability to exploit diverse habitats [14,15,16]. During the spread and outbreak, research has increasingly addressed its active and passive dispersal mechanisms, behavioral plasticity, and responses to environmental fluctuations, including changes in water level [17,18]. Although these studies have collectively provided substantial insight into the adaptive mechanisms of *P. canaliculata* across multiple biological levels and invasion stages, a comprehensive and systematic synthesis remains lacking.

Accordingly, this review aims to provide an integrated overview of the principal adaptive mechanisms underlying the invasion success of *P. canaliculata*. Specifically, we focus on: (1) its global distribution patterns and projected range expansion; (2) adaptive responses to abiotic stresses, including temperature, salinity, drought, and pollution; (3) strategies related to growth, reproduction, and resource utilization; (4) the roles of genomic plasticity, population genetic structure, and hybridization in invasion; (5) behavioral adaptations, including learning ability and alarm responses; and (6) Biological control of *P. canaliculata* and the global pet trade, as well as legislative restrictions on invasive *Pomacea* species. Through a systematic and detailed review, this article comprehensively elucidates the various synergistic factors that contribute to the successful invasion of *P. canaliculata*.

## 2. Materials and Methods

A structured literature review was conducted to summarize current knowledge on the invasion mechanisms of *P. canaliculata*. Literature was searched in Web of Science Core Collection, Scopus, PubMed, Google Scholar, and CNKI. Search terms included “*Pomacea canaliculata*”, “golden apple snail”, “apple snail” “invasive species”, “invasion ecology”, “physiological adaptation”, “phenotypic plasticity”, “molecular genetics”, “genomics”, “hybridization”, “introgression”, “behavior”, “cold tolerance”, “drought tolerance”, “salinity tolerance”, “pollution”, and “pesticide resistance”, “biological control”. Different combinations of these keywords were used to retrieve relevant publications.

The retrieved studies were screened according to their relevance to the major topics of this review, including physiological ecology, molecular mechanisms, behavioral adaptation, and invasion processes. Peer-reviewed articles in English and Chinese were prioritized. Additional references were identified by examining the reference lists and citations of key publications. This review is narrative rather than systematic, aiming to integrate findings across multiple scales to explain the invasion success of *P. canaliculata*.

## 3. Global and Regional Distribution Patterns of *P. canaliculata*

*Pomacea canaliculata* has expanded globally and is now recorded in multiple countries and regions across Africa, the Americas, Asia, Europe, and Oceania (Figure S1). In Africa, it has been reported from Kenya and Reunion. In the Americas, its distribution includes Argentina, Bolivia, Brazil, Chile, the Dominican Republic, Ecuador, Mexico, Paraguay, Peru, Trinidad and Tobago, the United States, and Uruguay. In Asia, the species is established in Cambodia, China, India, Indonesia, Israel, Japan, South Korea, Laos, Malaysia, Pakistan, the Philippines, Singapore, Thailand, and Vietnam. In Europe, it has been recorded in the Russian Federation, and in Oceania, it occurs in Guam and Papua New Guinea [19].

Species distribution models (SDMs) and environmental niche models (ENMs) indicate that many temperate, subtropical, and tropical regions worldwide are highly suitable for *P. canaliculata*. Although some of these areas have already been invaded, many large freshwater systems remain uncolonized but at high risk [20,21]. Under present climatic conditions, climatically suitable habitats occur on all continents. In addition to areas with confirmed occurrences, southern Africa, western Europe, and much of Oceania (except central Australia) also exhibit high habitat suitability [20,22] (Figure S2). Future climate projections suggest that climate change will generally facilitate further global expansion, although the effects are expected to be regionally heterogeneous. Suitable habitats are projected to contract in parts of South America, Africa, and Australia, but expand northward in Asia and North America and eastward in Europe. Additional range expansion is anticipated into higher-latitude regions, including northern Patagonia, the northern United States, and central Europe.

Climatic variables are major determinants of the distribution of *P. canaliculata*. Ensemble projections identify the minimum temperature of the coldest month as the primary factor limiting its northern range boundary [20]. In China, the temperature of the warmest quarter and the maximum temperature during the coldest months are key predictors of survival and growth [21]. Climate warming is likely to increase invasion risk by altering key physiological and ecological processes. Experimental evidence indicates that, near the current northern range limit, a 2 °C increase in temperature can raise annual feeding potential by approximately 21% [23]. Warming also enhances reproductive output, with egg production increasing fourfold, although individual growth may be slightly reduced, indicating a climate-dependent trade-off between growth and reproduction [24]. Cold-acclimation experiments across geographically distinct populations further show that *P. canaliculata* can survive prolonged exposure to 9–12 °C, with marked geographic variation in cold tolerance. These findings are consistent with predictions of northward and poleward range expansion under future warming scenarios [25].

Phylogenetic and molecular evidence suggests that invasive populations of *P. canaliculata* in China and other parts of Asia originated primarily from Argentina and were introduced through multiple independent events. These repeated introductions have facilitated rapid spread and helped maintain genetic diversity in the introduced range [26]. The species now forms extensive and stable populations across Asia, particularly in rice-growing areas of China, Japan, Korea, and Southeast Asia, where it occupies a variety of freshwater habitats, including croplands, rivers, ponds, wetlands, and paddy fields. These populations continue to threaten both agricultural production and aquatic ecosystems [27].

## 4. Multidimensional Tolerance and Adaptive Mechanisms to Abiotic Stress

**Figure 1:**
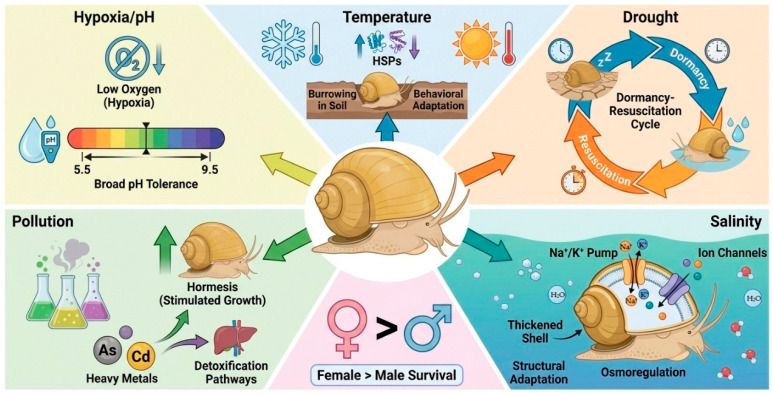
Multidimensional tolerance mechanisms of *Pomacea canaliculata*. This schematic illustrates that *P. canaliculata* can tolerate hypoxic conditions and survive across a pH range of 5.5–9.5. To cope with temperature fluctuations, the species employs behaviors such as burrowing and regulates heat shock protein expression. Under drought conditions, individuals rely on a dormancy–resuscitation cycle for survival. In response to salinity stress, they modulate ion transport systems and increase shell thickness. In addition, the species possesses effective detoxification pathways and exhibits toxic stimulation in response to heavy metal exposure. Females generally show higher stress tolerance than males.

*Pomacea canaliculata* exhibits remarkable multidimensional tolerance to a broad range of abiotic stresses, including extreme temperature, drought, salinity, chemical pollution, hypoxia, and pH fluctuations. Through coordinated physiological, molecular, and behavioral responses, the species persists in harsh and highly variable environments, thereby enhancing its invasive success and facilitating niche expansion (Figure 1).

### 4.1. Breaking Temperature Limits: Comprehensive Strategies from Molecular to Behavioral Levels

Temperature is a key factor limiting the distribution of *P. canaliculata*, particularly through winter minimum temperatures that define its northern range boundary. Nevertheless, the species employs a suite of coordinated adaptive mechanisms that mitigate this constraint (Table 1). *P. canaliculata* shows pronounced seasonal plasticity in cold tolerance. Individuals collected from summer rice fields are highly sensitive to low temperatures and survive no more than five days at 0 °C, whereas those collected in December from drained rice fields exhibit nearly 100% survival under the same conditions, indicating strong seasonal cold acclimatization [28]. This process is associated with gradual cooling and the accumulation of low-molecular-weight compounds, including glycerol, glutamine, and carnosine, together with reduced levels of glycogen and phenylalanine [29]. These changes suggest that *P. canaliculata* improves cold tolerance through compounds that function as cryoprotectants and/or osmotic regulators [30].

The apple snail also mitigates oxidative damage during cold exposure and hibernation by dynamically regulating antioxidant defenses and heat-shock proteins (HSPs) [34,35,36]. During hibernation, oxidative stress increases in the digestive gland, accompanied by elevated levels of uric acid, glutathione, and superoxide dismutase. Uric acid appears to play a particularly important protective role throughout the activity–hibernation cycle. Compared with non-invasive ampullariids such as *Lanistes nyassanus* and *Marisa cornuarietis*, *P. canaliculata* possesses a broader HSP gene repertoire [36]. Several of these genes are strongly induced by high temperatures, and tissue-specific expression patterns suggest that multiple HSPs contribute to resilience against cold-induced stress [34]. Cold acclimation also alters lncRNA and mRNA expression, affecting genes involved in proteasome function, linoleic acid metabolism, and retinol metabolism, thereby indicating extensive physiological reprogramming in support of cold tolerance [37].

At the behavioral level, *P. canaliculata* overwinters by burrowing into soil, particularly in paddy fields in southern China. Before winter, individuals burrow into the soil or hide beneath straw, thereby accessing microhabitats that remain above 0 °C even when surface temperatures fall below −5 °C [28]. After 120 days of hibernation, survival rates reach 73.6% in males, 87.5% in females, and 90.3% in juveniles, accompanied by metabolic suppression and increased antioxidant enzyme activity [31]. Cold tolerance is closely related to desiccation resistance; cold acclimation enhances both cold tolerance and survival under dry conditions [39]. Females generally exhibit greater cold resistance than males, with lower mean supercooling points and higher survival under combined cold–drought stress [30,31,38].

The thermal tolerance of *P. canaliculata* facilitates its spread into tropical and subtropical regions. Compared with non-invasive snails, it shows stronger resistance to temperature stress, and prolonged heat exposure induces marked changes in protein expression, indicating thermal adaptation [32,33]. Warmer conditions promote shell growth and delay female reproduction, while shifts in intestinal microbiota may also contribute to coping with extreme thermal stress [40].

### 4.2. Overcoming Drought: Exceptional Dormancy and Recovery Capabilities

Compared with the native species *Melanopsis tricarinata*, invasive *P. canaliculata* exhibits substantially higher drought tolerance [32]. This trait is expressed through a coordinated dormancy–resuscitation strategy. In response to drought, individuals actively burrow into the substrate and enter dormancy, a crucial behavioral adaptation (Table 2). Notably, females often deposit eggs before burrowing, thereby ensuring reproductive continuity [41]. During dormancy, *P. canaliculata* undergoes pronounced physiological and molecular adjustments that support survival. Transcriptomic analyses show downregulation of pathways associated with starch and sucrose metabolism and glutathione (GSH) metabolism. Immune-related pathways, including Toll-like receptor signaling and cell adhesion, are also suppressed [42]. This metabolic depression, together with transient immune downregulation, likely reduces energy expenditure while maintaining essential functions [42]. When favorable conditions return, the species exhibits strong recovery capacity, allowing rapid population re-establishment. Experimental studies show that more than 90% of surviving individuals resume normal activity, feeding, mating, and oviposition within 24 h after rehydration [41,43]. This rapid restoration of physiological and behavioral function is associated with the rapid reactivation of cell adhesion and immune-related pathways during resuscitation [42].

Notably, recent studies have revealed pronounced sexual dimorphism in drought adaptation mechanisms [41,43]. Females generally show higher survival under drought stress than males, as well as superior post-rehydration recovery, feeding performance, behavioral reactivation, and antioxidant responses [41]. This sex-specific difference may reflect divergent resource allocation strategies, with females having evolved more robust stress-tolerance mechanisms to safeguard reproductive investment. Moreover, drought tolerance appears to be physiologically associated with cold resistance, indicating a synergistic adaptative strategy that enhances the capacity to cope with multiple seasonal stressors [39].

### 4.3. Challenging Salinity Boundaries: Infiltration from Freshwater to Estuarine Environments

Although *P. canaliculata* is fundamentally a freshwater species, it exhibits a notable capacity to tolerate salinity and colonize brackish habitats (Table 3). It generally survives in waters with salinity below 5.0 practical salinity units (PSU), whereas survival declines markedly above 7.5 PSU; adults are typically more tolerant than juveniles [44,45,46,47]. Elevated salinity suppresses growth, feeding, and reproduction. Nevertheless, *P. canaliculata* can reinforce shell protection under saline stress by increasing calcium (Ca^2+^) deposition and shell protein synthesis, thereby enhancing shell strength, thickness, and width [44,46].

Transcriptomic evidence indicates that salinity exposure induces more differentially expressed genes (DEGs) in males than in females, suggesting that males may be more sensitive to salinity stress and experience greater physiological disturbance [53]. As in other aquatic organisms exposed to high salinity, *P. canaliculata* likely relies on the accumulation of organic osmolytes, such as glycerol and proline, to elevate intracellular osmotic pressure and reduce cellular dehydration [50]. In parallel, ion homeostasis is maintained through the regulation of ion channels and transporter-mediated fluxes of Na^+^, K^+^, and Ca^2+^ [51].

Salinity tolerance can be further enhanced by prior acclimation. Individuals preconditioned at relatively low salinities (e.g., 2–6 PSU) show higher tolerance thresholds and longer survival under subsequent exposure to higher salinities (e.g., 8–12 PSU) [44]. For instance, after acclimation at 6 PSU, some individuals can survive for up to 25 days at 8 PSU, demonstrating substantial physiological plasticity under gradual salinity increase. Field observations from Hong Kong provide additional support for this capacity, as wild *Pomacea* populations have been documented in naturally brackish habitats associated with shrimp collection and aquaculture. This salinity tolerance may facilitate invasion into estuarine and mangrove ecosystems, where *P. canaliculata* can survive and grow under simulated estuarine conditions, particularly at low to moderate salinity. Egg masses can also withstand periodic submergence, mimicking tidal cycles [48]. Under fluctuating salinity, *P. canaliculata* may enter a dormant state to conserve energy and resume activity and feeding when salinity declines [52]. In potential invasion habitats such as estuaries and mangroves, the species can also adjust its feeding strategy according to the physical and chemical properties of available vegetation, with intraspecific competition further influencing dietary preferences [49]. This flexibility implies a potential threat to certain mangrove plant species and highlights the ability of *P. canaliculata* to maintain trophic adaptability even under saline conditions.

### 4.4. Coping with Chemical Pollution: Tolerance and Stimulatory Effects

Chemical pollution constitutes a strong environmental filter that can asymmetrically alter competitive interactions between invasive and native species during introduction, establishment, and spread [3]. Available evidence indicates that *P. canaliculata* is more tolerant of a wide range of environmental pollutants than several native species (Table 4). With respect to heavy metals and metalloids, the median lethal concentrations (LC_50_) for trivalent and pentavalent arsenic in *P. canaliculata* are 12.63 and 18.62 mg/kg, respectively, both higher than those reported for native species such as *Bellamya quadrata* and *Cipangopaludina cathayensis*. Physiologically based pharmacokinetic (PBPK) modeling further suggests that *P. canaliculata* has a lower arsenic accumulation capacity and higher metabolic efficiency. Subcellular distribution analyses indicate that arsenic is preferentially sequestered into detoxification-related compartments, which likely contributes to its high arsenic tolerance [54]. The species also exhibits substantially greater cadmium tolerance than native snails, with 48-, 72-, and 96 h LC_50_ values of 4.26, 2.24, and 1.98 mg/L, respectively—approximately three times those of native counterparts [55].

*Pomacea canaliculata* also shows considerable resistance to multiple pesticides. Acute toxicity assays have demonstrated high tolerance to spirotetramat (STM) and cypermethrin (CYP). Compared with other tested species, *P. canaliculata* is the most CYP-tolerant mollusk and the most STM-tolerant freshwater organism reported to date [18]. It also exhibits resistance to molluscicides and pesticides such as metaldehyde and niclosamide ethanolamine salt, potentially associated with the large repertoire of cytochrome P450 genes identified in its transcriptome [58]. In addition, the species shows substantial tolerance to low concentrations of glyphosate (0.5–2 mg/L) [56,57]. Field surveys further indicate lower tributyltin bioaccumulation in *P. canaliculata* than in the aquaculture clam *Corbicula fluminea*, suggesting stronger homeostatic regulation under polluted conditions [62].

Notably, exposure to low pollutant concentrations often elicits stimulatory rather than inhibitory effects in *P. canaliculata*, consistent with hormesis. Such responses may enhance growth, feeding, and physiological repair capacity. For example, exposure to 20 μg/L copper increases feeding rate by 28%, whereas 30 μg/L promotes growth [59]. Low arsenic concentrations not only stimulate growth but also upregulate genes involved in DNA replication and chitin synthesis, while increasing gut microbiota diversity and promoting arsenic biotransformation [60,63]. Similarly, exposure to 2 mg/L glyphosate improves growth performance, increases oviposition, and elevates estrogen levels [56,57]. Exposure to 20 μg/L of microplastics, although disruptive to biomineralization pathways, unexpectedly enhances shell repair potential [61]. Collectively, these findings indicate that *P. canaliculata* can modulate physiological state and activate protective mechanisms under sublethal pollution stress, thereby increasing resilience and invasion potential in contaminated habitats.

### 4.5. Tolerance to Other Abiotic Stressors

In addition to temperature, drought, salinity, and pollutants, *P. canaliculata* shows high tolerance to other environmental stressors, notably hypoxia and pH fluctuation. Compared with its non-invasive congener *P. diffusa*, *P. canaliculata* exhibits stronger resistance to hypoxic stress. This advantage appears to be mediated by molecular responses involving downregulation of energy-intensive pathways such as glycolysis, together with upregulation of signaling pathways that help maintain cellular homeostasis. Several related genes also show evidence of positive selection, suggesting a genetic basis for this tolerance [13]. Relative to native snails such as *Pila scutata*, *P. canaliculata* can maintain normal feeding, growth, and high survival under conditions of extremely low dissolved oxygen and severe organic pollution, whereas *P. scutata* exhibits growth inhibition or mortality under the same conditions [12].

The species also tolerates a broad pH range. Both short- and medium-term experiments, including 28-day exposure studies, show that *P. canaliculata* can survive at pH 5.5–9.5 without significant effects on survival [64]. This broad acid–base tolerance indicates a strong capacity to withstand common fluctuations in water chemistry. Together, these multifaceted tolerances enable *P. canaliculata* to colonize and dominate degraded habitats that are unsuitable for many native species, thereby enhancing its competitive advantage, expanding its ecological niche, and increasing its invasion potential.

## 5. Adaptive Strategies for Growth, Reproduction, and Resource Utilization

**Figure 2:**
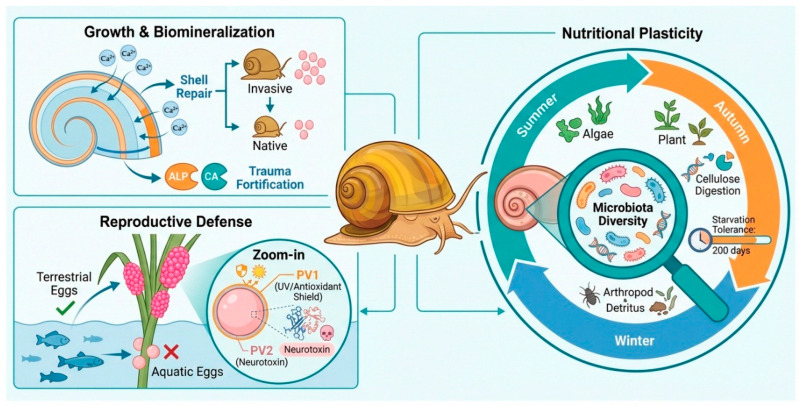
Adaptive strategies of *Pomacea canaliculata* for growth, reproduction, and resource utilization. This schematic summarizes three major survival-related traits of *P. canaliculata*. For growth, the species relies on the coordinated action of Ca^2+^ and shell-related enzymes to repair and reinforce shell damage. Nutritionally, it responds to seasonal variation through flexible consumption of algae and vascular plants, uses gut microbiota to facilitate digestion, and can withstand prolonged starvation for up to 200 days. Reproductively, it preferentially deposits eggs above the water surface to reduce aquatic predation. The eggs are protected by perivitellin-1 (PV1), which provides ultraviolet protection and antioxidant defense, and perivitellin-2 (PV2), which functions as a neurotoxic antipredator factor.

*Pomacea canaliculata* exhibits highly plastic growth, flexible feeding strategies, and a distinctive reproductive defense system that together promote rapid establishment, efficient resource acquisition, and sustained population expansion. These life-history traits are central to its invasion success across diverse freshwater habitats (Figure 2).

### 5.1. Growth Strategies of P. canaliculata

*Pomacea canaliculata* exhibits marked adaptability and plasticity in growth-related traits, including rapid individual growth, high reproductive potential, and exceptional shell repair and biomineralization capacity (Table 5). Populations from invaded regions such as China show faster shell growth, earlier sexual maturation, higher fecundity, and greater hatching success than populations from the native range in Argentina when reared under comparable environmental conditions [65]. This apparent rapid adaptive divergence is likely driven by strong selective pressures in invaded habitats, including irrigation regimes in rice fields and repeated pest-control disturbance, which may favor enhanced population growth in novel environments. Temperature strongly influences growth performance in *P. canaliculata*. Individuals reared at higher temperatures exhibit faster shell elongation and develop mechanically stronger shells [66]. Environmental Ca^2+^ availability is also critical for shell development and structural integrity. Sufficient Ca^2+^ promotes biomineralization and results in thicker, harder shells [67]. *Pomacea canaliculata* can efficiently utilize Ca^2+^ from both water and food to repair shell damage through enhanced biomineralization, thereby maintaining structural integrity and defensive capacity [68].

The species also possesses strong shell regenerative ability. Following partial shell removal, survival can exceed 90%, with repair initiating within a few days and typically completing within 1–2 weeks. This process is accompanied by significant increases in the activities of key biomineralization-related enzymes, including alkaline phosphatase (ALP) and carbonic anhydrase (CA), as well as a transient increase in circulating hemocytes, indicating coordinated mobilization of physiological and cellular resources for shell reconstruction [69]. Repeated cycles of shell damage and repair may induce shell thickening, suggesting a trauma-induced fortification effect. Although repair efficiency can vary among shell color morphs, overall regenerative capacity remains high [70]. These growth-related traits underscore the strong ecological resilience of *P. canaliculata* and contribute to its success as an invasive species.

### 5.2. Flexible Nutrition and Feeding Strategies

*Pomacea canaliculata* displays substantial flexibility in feeding and nutritional strategies, which is a key determinant of its ability to persist under variable environmental conditions and successfully invade new habitats (Table 6). Its trophic strategy includes efficient resource acquisition, broad dietary breadth, seasonal and habitat-dependent diet shifts, and alternative feeding behaviors under resource limitation. In addition, the species can exert suppressive effects on co-occurring native snails. Compared with local snail species such as *P. angelica*, *P. canaliculata* exhibits higher feeding rates both in isolation and in mixed-species settings, indicating strong exploitative competitive ability [71]. Beyond direct food competition, it can negatively affect many native and non-native snail species through physical contact or close proximity, thereby altering community structure [72]. These findings suggest that the invasion success of *P. canaliculata* depends not only on resource competition but also on interference interactions that further reinforce its ecological dominance.

The diet of *P. canaliculata* varies markedly across seasons and habitat types, reflecting pronounced trophic plasticity. Stable isotope and DNA metabarcoding analyses indicate seasonal dietary shifts, including preference for filamentous algae in summer, increased consumption of vascular plants in autumn, and ingestion of arthropods in winter. Dietary composition also differs among aquatic habitats such as ponds, rivers, and ditches [14,15,16]. These diet shifts directly influence gut microbiota composition and function, which vary substantially among habitats and are primarily shaped by food resource availability [15]. Changes in diet affect microbial community structure and associated metabolic pathways, including amino acid biosynthesis, thereby supporting physiological adjustment to fluctuating environments [16]. This linked cascade—from diet to microbiome to metabolic regulation—provides an important mechanistic basis for the invasion success of the species.

The gut metagenome of *P. canaliculata* is enriched in genes encoding diverse carbohydrate-active enzymes, which enhance its capacity to utilize a wide range of plant-derived foods, including cellulose-rich material [76]. When fresh plant resources are scarce, the species can also ingest sediment to obtain organic matter, a strategy that helps juveniles prolong survival and maintain growth [73]. This behavior not only enhances persistence during food limitation but may also alter biogeochemical cycling by reducing sediment organic matter, reinforcing its role as an ecosystem engineer. Relative to many other gastropods, *P. canaliculata* also shows pronounced starvation tolerance [74]. Juveniles survive on average up to 52.6 days without food, whereas some adults can survive for more than 200 days, a trait that likely facilitates dispersal and establishment in newly invaded habitats [75]. Under food shortage, reproductive investment can be rapidly reduced, with egg laying suspended to conserve energy and resumed quickly once food becomes available, indicating adaptive allocation under fluctuating resource conditions [75].

### 5.3. Unique Reproductive Defense System

*Pomacea canaliculata* has exceptionally high reproductive output, which is a major contributor to its invasion success and capacity to form dominant populations (Table 7). A single female can produce approximately 13,764 eggs over its lifetime, resulting in about 6070 hatchlings, far exceeding the reproductive output of most native snails and bivalves [77]. However, high fecundity alone does not fully explain its success. The species also possesses a multilayered reproductive defense system that enhances the survival of eggs and juveniles, thereby providing dual protection during invasion and establishment.

Eggs are deposited on emergent terrestrial substrates above the waterline, including shoreline structures and plant stems, where they form tightly packed, carotenoid-rich masses. This oviposition behavior reduces exposure to fish, crustaceans, aquatic invertebrate predators, and parasites, and thus represents a primary defense strategy based on spatial separation from aquatic enemies. In contrast to the aquatic egg-laying species *Marisa cornuarietis*, the terrestrial egg-laying species *P. canaliculata* and *P. maculata* possess perivitelline fluid (PVF) components such as PV2, a neurotoxic protein lethal to mice, as well as a calcium-binding protein potentially involved in eggshell calcification [78].

PVF is a key biochemical component of this reproductive defense system. It is composed predominantly of two multifunctional glycoprotein complexes, PV1 and PV2, which together account for 80–85% of total PVF protein [79]. Multi-omics analyses and functional experiments indicate that these proteins contribute not only to embryo nutrition but also to multiple defensive functions [76]. PV1 is a highly stable yolk-like glycoprotein complex characterized by strong structural resistance to degradation and stability across a broad pH range and under proteolytic conditions [83,84,85]. Associated carotenoids contribute warning coloration to egg masses and provide antioxidant and ultraviolet protection, thereby safeguarding embryos developing in exposed terrestrial environments [86,87]. Evolutionary analyses suggest that, during the phylogenetic diversification of *Pomacea*, PV1 underwent a trade-off between structural stability and ecological function, retaining substantial robustness while gaining stronger warning coloration and antinutritive properties that may enhance invasive success [89]. PV2 is the principal toxic component of PVF. It is a neurotoxic protein formed by covalent linkage between membrane attack complex/perforin-like domains and lectin-like domains, and is highly toxic to vertebrates, with an oral median lethal dose (LD_50_) in mice of approximately 5–6 mg/kg [80]. Egg extracts also exhibit acute neurotoxicity in amphibians and inhibit key enzymes such as acetylcholinesterase [81]. More recent studies further show that PVF affects invertebrate predators, including mealworm beetle larvae, causing mortality after injection and physiological damage after oral ingestion, along with delayed development and reduced reproduction [82].

Although these defensive proteins deter many external predators, they exert limited effects on conspecifics, which may permit occasional cannibalism. Under food limitation, eggs or egg remains can serve as emergency nutrition for juveniles or adults, creating an internal nutrient recycling pathway [88]. Overall, the combination of extremely high fecundity and an integrated, multilayered reproductive defense system not only secures propagule supply but also substantially increases offspring survival. This synergistic strategy likely explains the low predation rates on eggs in natural habitats and provides a critical foundation for rapid establishment and long-term population persistence during global invasion.

## 6. Genetic and Evolutionary Foundations: Genomic Plasticity and Hybridization

### 6.1. Invasive Adaptation at the Genomic Level

The invasion success of *P*. *canaliculata* is closely associated with its adaptive genomic architecture. Whole-genome sequencing has revealed a genome of approximately 440 Mb containing recently expanded DNA/hAT-Charlie transposable elements. These elements likely contribute to elevated genetic variation and rapid evolutionary responses, thereby enhancing phenotypic plasticity under environmental stress [76]. In addition, marked expansion of the cytochrome P450 gene family has likely strengthened the capacity of *P. canaliculata* to metabolize and detoxify xenobiotics, improving tolerance to pollutants and pesticide exposure. Genes involved in stress resistance also appear to have undergone adaptive evolution. For example, the cold shock protein-coding gene *CSDE1*, identified as a target of natural selection, is significantly upregulated under cold stress, providing a molecular basis for enhanced cold tolerance [90]. Moreover, the gut metagenome of *P. canaliculata* is enriched in carbohydrate-active enzyme genes, which likely facilitate the efficient digestion of diverse plant-derived substrates and broaden dietary flexibility [76]. Collectively, these genomic features provide an intrinsic molecular toolkit that enables rapid adaptation to heterogeneous and challenging environments.

### 6.2. Population Genetic Dynamics and Hybridization

Asian populations of *P. canaliculata* exhibit high genetic diversity and pronounced population differentiation, largely reflecting multiple independent introduction events and complex invasion histories [91,92]. Genetic variation is more strongly partitioned among populations than within populations and displays clear geographic structuring. Some genetic discontinuities coincide with major geographic features, such as the Yangtze River basin and national borders, suggesting that both climatic constraints and human-mediated dispersal have shaped current distribution patterns [90,92]. Hybridization with closely related species, particularly *P. maculata*, has further influenced the evolutionary trajectory of *P. canaliculata* during invasion [93,94]. Genetic analyses from China indicate that pure homozygous genotypes of *P. maculata* have become exceedingly rare, whereas hybrid individuals are now widespread. These populations often show cytonuclear discordance, with *P. canaliculata* mitochondrial haplotypes co-occurring with admixed nuclear backgrounds. Such introgression may generate novel trait combinations, including the cold tolerance of *P. canaliculata* and the larger body size of *P. maculata*, thereby promoting hybrid vigor and increasing invasion potential [94]. Hybridization may also homogenize reproductive traits, such as egg size and clutch characteristics, while altering parental population dynamics through competitive displacement. The resulting genetically complex hybrid populations may be especially difficult to detect and manage. In colder regions of Asia, the lower cold tolerance of *P. maculata* may limit its persistence; however, hybrid individuals that acquire enhanced cold tolerance may survive and expand. These patterns underscore the important role of hybridization in facilitating adaptive divergence and range expansion during biological invasion [94].

## 7. Learning Behavior and Alarm Response

Beyond its strong physiological adaptability, *P. canaliculata* exhibits considerable behavioral flexibility that further enhances survival and invasion success in novel environments. One particularly important adaptation is its capacity to recognize predator-associated cues and respond appropriately to alarm signals. Experimental evidence indicates that *P. canaliculata* can acquire predator recognition through associative learning. Juvenile snails exposed simultaneously to injured conspecifics (alarm cues) and live predators, such as carp or turtles, subsequently exhibit stronger defensive responses to those same predators even in the absence of alarm cues. These responses, including climbing out of the water, are significantly more pronounced than those of untrained individuals. Notably, this learning is predator-specific, indicating that the species can discriminate among threats and adjust its antipredator behavior accordingly [17].

*Pomacea canaliculata* also displays characteristic alarm responses to chemical cues released by injured conspecifics, including emersion and burrowing into sediment [95]. The intensity of these responses in natural settings is influenced by habitat structure. For example, alarm responses are more frequent in densely vegetated habitats than in sparsely vegetated ones, possibly because vegetation provides greater physical refuge and thereby facilitates escape behavior. At the same time, *P. canaliculata* exhibits a clear trade-off between predator avoidance and resource acquisition. Although individuals respond defensively to alarm cues, they may also consume injured conspecifics, reflecting opportunistic foraging under risk. This balance between antipredator behavior and nutritional gain highlights the behavioral plasticity that contributes to the invasive success of this species [95].

## 8. Biological Control of *P. canaliculata*

In terms of biological control of *P. canaliculata*, domestic research and practice focus on the exploration of native natural enemy resources and the integration of ecological planting and breeding models, forming a technical path with both snail-control effects and economic value. *Whitmania pigra* has no preference for the size of *P. canaliculata* prey and can inhibit its growth and development through direct predation and predation risk [96]. Juvenile *Eriocheir sinensis* prefer to feed on small-sized *P. canaliculata* and can improve their own nutritional quality while achieving the goal of snail control [97]. The suitable release density of *Pelodiscus sinensis* in *Zizania latifolia* fields is 30~50 individuals per 666.7 m^2^, which has a significant snail control effect and has been widely adopted by farmers [98]. Ecological models such as rice-duck symbiosis, rice-prawn symbiosis (*Macrobrachium rosenbergii*), and rice-carp culture can all effectively inhibit the population of *P. canaliculata*. Among these, the recovery rate of *Cyprinus carpio* in rice fields reaches 90%, and the recommended snail control density is 2041 individuals per hectare [99,100,101]. *Tetraodon nigroviridis* has a significantly higher predation efficiency on *P. canaliculata* in brackish water than in freshwater [102]. *Chinemys reevesii* has extremely strong predation ability, with a single individual able to prey on more than 2000 *P. canaliculata* within 8 weeks [103]. It should be noted that although *Mylopharyngodon piceus* and *C. carpio* have snail-control effects, their potential impacts on non-target animals and plants need to be paid attention to in application [104,105]. This type of control method can effectively control the population size of *P. canaliculata* and achieve a win-win situation between ecological and economic values. Internationally, different regions have carried out targeted control practices relying on local natural enemy resources. *Corvus corone* Linnaeus in Japan has strong predation ability on *P. canaliculata* in rice fields, with a predation rate as high as 87.1% within 1.2 m of the levee in Ehime Prefecture within 2 days [106]. The native *Anabas testudineus* in Laos can significantly reduce the number of juvenile snails, while *Esanthelphusa nimoafi* can prey on *P. canaliculata* of all sizes [107]. In North America, ants of the genus *Crematogaster* have been found to prey on *P. canaliculata* eggs, providing a new natural enemy resource for local *P. canaliculata* control [108]. In summary, there are various natural enemies of *P. canaliculata* worldwide, including annelids, arthropods, fish, reptiles, and birds. Both domestic and international regions have formed targeted biological control technical paths combined with local resources, providing effective support for the prevention and control of *P. canaliculata* invasion.

## 9. Global Pet Trade and Legislative Restrictions of Invasive *Pomacea* Species

Although global trade volume data for apple snails remain incomplete, regional surveys indicate substantial circulation in the ornamental pet trade. In South Africa, for instance, *P. canaliculata* was recorded in 74 out of 117 surveyed pet stores, with large numbers of individuals sold year-round, peaking in summer [109]. Similar patterns have been documented in Europe, Asia, and North America, where apple snails are among the most commonly traded freshwater gastropods [110,111,112]. Widespread availability, high market demand, and low prices collectively drive large-scale transnational transport, creating considerable propagule pressure and invasion risk via unintentional release or escape.

Stringent legislative restrictions have been implemented in Europe to reduce invasion risks from traded apple snails. Many *Pomacea* species are listed as invasive alien species of Union concern under EU Regulation 1143/2014, which prohibits their import, trade, possession, and release into the environment. Similar regulatory frameworks exist in North America and parts of Asia, targeting the prevention of human-mediated dispersal via the pet trade. However, enforcement gaps and online trade continue to challenge effective management, highlighting the need for coordinated monitoring and stricter cross-border biosecurity.

## 10. Conclusions

The invasiveness of *P. canaliculata* serves as a model of synergistic factors at multiple levels, highlighting a powerful invasion syndrome. Its success is attributed to genomic plasticity, characterized by transposon bursts and expansions of the gene family, and rapid evolution through mechanisms such as natural selection and hybridization. These intrinsic mechanisms provide resilience against multiple environmental stressors, including temperature fluctuations, drought, salinity, and pollution. Complementing these defenses, its high reproductive output, protected eggs, flexible resource utilization through generalist feeding and detritivore, and robust shell regenerative capabilities act as offensive strategies for expansion. These strategies allow for continued exploration of new territories within diffusion pathways influenced by human activities such as rice trade and aquaculture. Future research should focus on the following directions:

Integration of multiomics data: Future research should utilize transcriptomics, proteomics, and metabolomics to build a comprehensive regulatory network linking genes to phenotypes. This approach will provide deeper insights into the adaptive mechanisms of *P. canaliculata* and improve predictions of its evolutionary responses to environmental changes.

Ecological and evolutionary consequences of hybridization: Long-term studies of hybrid populations are crucial for understanding whether hybridization improves invasiveness or leads to reduced fitness. This research will clarify the role of hybridization in the invasion process and inform management strategies.

Assessment of composite stress effects: Investigating how multiple stressors, such as temperature rise, eutrophication, pollution, and salinity, interact is critical to predicting population dynamics and distribution shifts. Such insights are essential for modeling future invasion scenarios under changing environmental conditions.

Development of precision control technologies: Environmentally friendly strategies, such as RNA interference and targeted inhibitors, should be developed to disrupt critical metabolic or reproductive pathways. These precision approaches can effectively manage the populations of *P. canaliculata* while minimizing ecological impacts.

## Figures and Tables

**Table 1:** Adaptive traits of *Pomacea canaliculata* in response to temperature stress.

Stress Type	Tolerance Range/Key Physiological Indicators	Core Adaptation Mechanisms	Sexual Dimorphism	Key Adaptive Characteristics and Invasion Advantages
Temperature Stress (Low/High Temperature)	1. Low temperature: Can survive long-term at 0 °C after acclimatization; the microenvironment in soil for natural overwintering is above 0 °C, and can survive when the surface temperature drops to −5 °C [28]. 2. Lower supercooling point; survival rate after 120 days of overwintering: 73.6% for males, 87.5% for females, and 90.3% for juveniles [31]. 3. High temperature: Heat tolerance is significantly higher than that of native snails, and physiological adaptation can be completed under long-term heat stress [32,33].	1. Accumulation of small-molecule osmoprotectants (glycerol, glutamine, carnosine, etc.) [29,30]. 2. Regulation of antioxidant system and heat shock proteins (HSPs) [34,35,36]. 3. Reprogramming of lncRNAs and mRNAs, remodeling lipid and vitamin metabolism [37]. 4. Behavioral dormancy and burrowing for overwintering [28,31].	Females have significantly better cold tolerance and cold-drought stress resistance than males [30,31,38].	Strong seasonal acclimatization ability; broad temperature adaptation supports north–south expansion and colonization in tropical and subtropical regions [28,32,33].

**Table 2:** Adaptive traits of *P. canaliculata* in response to drought stress.

Stress Type	Tolerance Range/Key Physiological Indicators	Core Adaptation Mechanisms	Sexual Dimorphism	Key Adaptive Characteristics and Invasion Advantages
Drought Stress	Can survive long-term drought dormancy and quickly restore physiological activities after rehydration; more than 90% of surviving individuals resume normal activities within 24 h of rehydration [41,43].	1. Actively burrow into the substrate to enter dormancy, inhibiting starch, sucrose, and glutathione metabolism [41,42]. 2. Downregulate immune pathways to reduce energy consumption [42]. 3. Rapidly activate adhesion and immune pathways after rehydration for rapid repair [42].	Females have a higher drought survival rate and better feeding and antioxidant repair abilities after rehydration [41,43].	Improved dormancy-resuscitation system; metabolic suppression saves energy, and rapid population reconstruction can be achieved with short-term water supplement [41,42,43].

**Table 3:** Adaptive traits of *P. canaliculata* in response to salinity stress.

Stress Type	Tolerance Range/Key Physiological Indicators	Core Adaptation Mechanisms	Sexual Dimorphism	Key Adaptive Characteristics and Invasion Advantages
Salinity Stress	1. Normal tolerance salinity ≤ 5.0 PSU; survival is limited when salinity exceeds 7.5 PSU, and adults have higher tolerance than juveniles [44,45,46,47]. 2. After acclimatization to low salinity (2–6 PSU), it can tolerate high salinity environments (8–12 PSU) [44]. 3. Can grow and reproduce normally in low to moderate salinity environments such as estuaries and mangroves [48,49].	1. Increase Ca^2+^ accumulation and shell protein synthesis to thicken and widen the shell [44,46]. 2. Synthesize osmoprotectants such as glycerol and proline [50]. 3. Regulate Na^+^/K^+^/Ca^2+^ homeostasis through ion channels and transport proteins [51]. 4. Can enter dormancy to save energy under high salinity [52].	Males have more differentially expressed genes under salinity stress and are more sensitive to salinity [53].	Can invade estuaries and mangroves at the junction of salt and fresh water; strong plasticity under salinity fluctuations, and feeding habits can be adjusted with habitats [48,49,52].

**Table 4:** Adaptive traits of *P. canaliculata* in response to chemical pollution stress.

Stress Type	Tolerance Range/Key Physiological Indicators	Core Adaptation Mechanisms	Sexual Dimorphism	Key Adaptive Characteristics and Invasion Advantages
Chemical Pollution Stress (Heavy Metals/Pesticides/Pollutants)	1. Arsenic: LC_50_ for trivalent arsenic is 12.63 mg/kg, and LC_50_ for pentavalent arsenic is 18.62 mg/kg [54]. 2. Cadmium: 48/72/96 h-LC_50_ are 4.26, 2.24, and 1.98 mg/L, respectively [55]. 3. Tolerant to various pesticides such as spirotetramat, cypermethrin, and glyphosate (low concentration 0.5–2 mg/L) [18,56,57].	1. Subcellular compartment sequestration of pollutants, low accumulation, and efficient metabolic detoxification [54,55]. 2. Expansion of cytochrome P450 family genes to enhance pesticide metabolism [58]. 3. Continuous activation of antioxidant and detoxification pathways [54,55,59].	No significant sexual dimorphism in pollution tolerance was reported in the original text.	Low-concentration pollutants exhibit “hormesis effect”, promoting growth, feeding, reproduction, and shell repair; strong competitive advantage in polluted habitats [56,57,59,60,61].

**Table 5:** Growth strategies, key mechanisms, and adaptive advantages of *P. canalicula*.

Adaptive Strategy Type	Key Indicators/Characteristics	Core Mechanisms	Adaptive Advantages
Growth Strategies	1. Invasive populations have faster shell growth, earlier sexual maturity, higher fecundity, and hatching success than native populations [65]. 2. High temperature and sufficient Ca^2+^ promote shell growth and hardness [66,67]. 3. Shell repair survival rate > 90%, repair completes in 1-2 weeks; multiple repair cycles lead to thicker shells [69,70].	1. Rapid adaptive evolution driven by invasion-related selective pressures (e.g., rice paddy irrigation, pest control) [65]. 2. Utilize Ca^2+^ from water and food for biomineralization [67,68]. 3. Increased activity of ALP and CA, and temporary elevation of circulating hemocytes during shell regeneration [69].	Enhances adaptability to diverse environments; maintains structural integrity and defense capabilities; promotes rapid population expansion [65,69,70].

**Table 6:** Flexible nutrition and feeding strategies, core mechanisms, and adaptive advantages of *P. canaliculata*.

Adaptive Strategy Type	Key Indicators/Characteristics	Core Mechanisms	Adaptive Advantages
Flexible Nutrition and Feeding Strategies	1. Higher feeding rate than native snails; exerts inhibitory effects on coexisting local snail species [71,72]. 2. Seasonal-habitat-driven dietary shifts; consumes algae, vascular plants, arthropods, and sediment (when food is scarce) [14,15,16,73]. 3. Strong starvation tolerance: juveniles survive ~52.6 days, adults survive > 200 days [74,75].	1. Rich in gut microbial genes encoding carbohydrate-active enzymes (e.g., cellulose-degrading enzymes) [76]. 2. Dietary shifts regulate gut microbiota and metabolic pathways (e.g., amino acid biosynthesis) [15,16]. 3. Adjusts reproductive investment to conserve energy during food scarcity [75].	Facilitates adaptation to changing food resources; strengthens ecological competitiveness; supports survival and dispersal in resource-scarce habitats [14,15,16,71,72,73,74,75,76].

**Table 7:** Unique reproductive defense system, core mechanisms, and adaptive advantages of *P. canaliculata*.

Adaptive Strategy Type	Key Indicators/Characteristics	Core Mechanisms	Adaptive Advantages
Unique Reproductive Defense System	1. High fecundity: a single female lays ~13,764 eggs in her lifetime, with ~6070 hatchlings [77]. 2. Lays terrestrial egg masses; PVF contains defensive proteins (PV1, PV2) [78,79]. 3. PV2 is a neurotoxin (LD_50_ for mice: 5–6 mg/kg); egg extracts are toxic to amphibians and invertebrates [80,81,82].	1. Terrestrial egg-laying reduces predation by aquatic organisms [78]. 2. PV1 provides structural stability, UV and antioxidant protection; carotenoids offer warning coloration [83,84,85,86,87]. 3. PV2 deters vertebrate and invertebrate predators; egg cannibalism provides emergency nutrition [80,82,88].	Ensures high offspring survival rate; secures population base; supports rapid establishment and stability during global invasion [77,79,80,88,89].

## Data Availability

No new data were created or analyzed in this study.

## Supplementary Materials

The following supporting information can be downloaded at: https://www.mdpi.com/article/10.3390/biology15100747/s1, Figure S1 Current global distribution of *Pomacea canaliculata* (modified from the EPPO Global Database map, SVG format). https://gd.eppo.int/taxon/POMACA/distribution (accessed on 5 January 2026). Figure S2 Global predictions of currently suitable areas for *P. canaliculata* based on ENM.

## Abbreviations

The following abbreviations are used in this manuscript:

ALP	Alkaline phosphatase
CA	Carbonic anhydrase
CYP	Cypermethrin
DEGs	Differentially expressed genes
ENMs	Environmental niche models
GSH	Glutathione
HSPs	Heat-shock proteins
IUCN	International Union for Conservation of Nature
LC_50_	Median lethal concentrations
LD_50_	Median lethal dose
PBPK	Physiologically based pharmacokinetic
PSU	Practical salinity units
PV1	Perivitellin-1
PV2	Perivitellin-2
PVF	Perivitelline fluid
SDMs	Species distribution models
STM	Spirotetramat
