# Supplementary material for: Multiscale Mechanisms Underlying the Invasion Success of Pomacea canaliculata: A Review
Source: Biology (Basel). 2026 May 8;15(10):747. doi: 10.3390/biology15100747 (PMC13203566; doi:10.3390/biology15100747)
Supplement: Supplementary file 1 [file biology-15-00747-s001.zip › biology-4299480-supplementary.pdf]

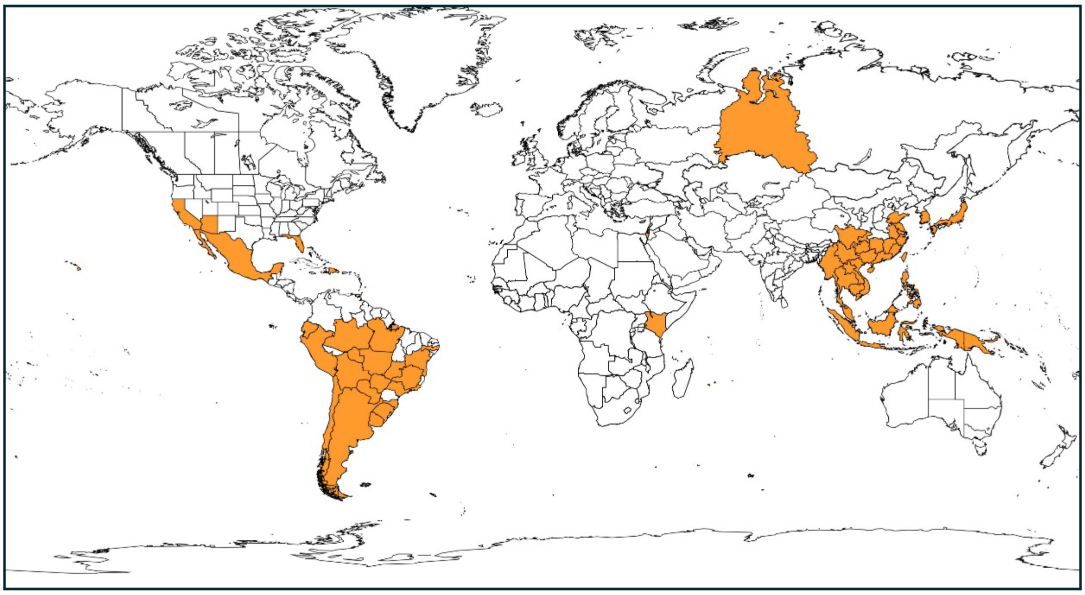

Figure S1. Current global distribution of *Pomacea canaliculata* (modified from the EPPO Global Database map, SVG format). https://gd.eppo.int/taxon/POMACA/distribution (accessed on 5 January 2026).

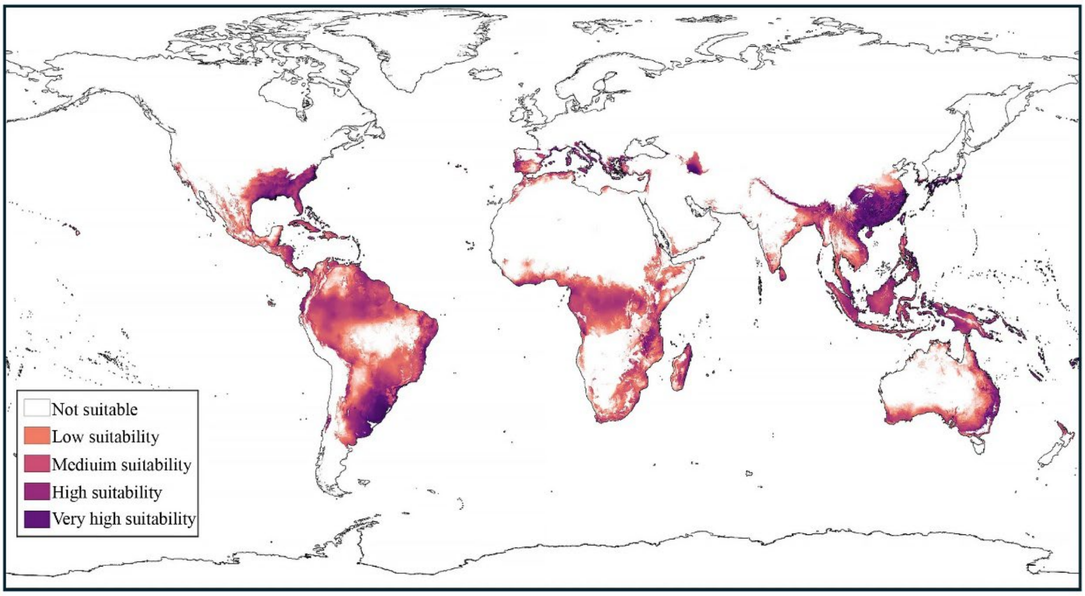

Figure S2. Global predictions of currently suitable areas for *P. canaliculata* based on ENM.
